# Differential effects of face-realism and emotion on event-related brain potentials and their implications for the uncanny valley theory

**DOI:** 10.1038/srep45003

**Published:** 2017-03-23

**Authors:** Sebastian Schindler, Eduard Zell, Mario Botsch, Johanna Kissler

**Affiliations:** 1 Department of Psychology, Bielefeld University, Germany; 2 Computer Graphics Group, Bielefeld University, Germany

## Abstract

Cartoon characters are omnipresent in popular media. While few studies have scientifically investigated their processing, in computer graphics, efforts are made to increase realism. Yet, close approximations of reality have been suggested to evoke sometimes a feeling of eeriness, the “uncanny valley” effect. Here, we used high-density electroencephalography to investigate brain responses to professionally stylized happy, angry, and neutral character faces. We employed six face-stylization levels varying from abstract to realistic and investigated the N170, early posterior negativity (EPN), and late positive potential (LPP) event-related components. The face-specific N170 showed a u-shaped modulation, with stronger reactions towards both most abstract and most realistic compared to medium-stylized faces. For abstract faces, N170 was generated more occipitally than for real faces, implying stronger reliance on structural processing. Although emotional faces elicited highest amplitudes on both N170 and EPN, on the N170 realism and expression interacted. Finally, LPP increased linearly with face realism, reflecting activity increase in visual and parietal cortex for more realistic faces. Results reveal differential effects of face stylization on distinct face processing stages and suggest a perceptual basis to the uncanny valley hypothesis. They are discussed in relation to face perception, media design, and computer graphics.

The face is perhaps the most powerful communication channel for human social interaction, enabling the observer to quickly infer information about the sender^1^,^2^. Faces allow not only recognizing a unique identity, but also communicating basic emotional states. Dating back to Darwin^3^, faces and facial expressions have been extensively studied in psychology and neuroscience, resulting in elaborate cognitive^4^ and neuroscientific theories (e.g. see refs 5 and 6). Some theories also had a big impact on other disciplines. Ekman’s theory of universal basic emotions^7^,^8^ and the resulting Facial Action Coding System (FACS) became very popular in computer graphics and are a fundamental concept in current facial animation systems. By blending between different FACS expressions a complex facial expression is created for a virtual character.

In this regard, advancements in face perception research are also of high interest for many commercial productions in the game or visual effects industry. Convincing characters highly contribute to the story and visual quality of a movie binding a lot of time and money at production time. In the movie “Maleficent” for example, expensive hardware, custom software, and months of work by highly skilled professionals and researchers was required to create the digital faces of the flower pixies^9^. However, little is known about how stylized faces are processed by the brain and about which facial details are important in particular. Even less is known about how a presented emotional expression interacts with stylization of a face. Concerns about the influence of stylization have recently also become an issue for face perception research in psychology, where computer generated characters start to replace photographs of real people due to higher flexibility and better experimental stimulus control^10^.

Regarding the effect of character realism on human perception, so far, the uncanny valley theory^11^ dominates the discourse in research and practice. According to Mori, humanoids are in general perceived as more appealing if they are more realistic. However, humanoids that are more realistic than cartoon characters, but do not fully achieve human-likeness, may cause an “eerie feeling” and fall into the “uncanny valley”. This theory has been developed on the basis of Mori’s personal observation in the 1970s and has been subject of some empirical research in the last decade. Initial research used morphs of different characters^12^ or videos of different robots at different stylization levels^13^. Also image editing algorithms^14^ or different rendering algorithms^15^ have been applied to create facial stimuli at different stylization levels. Other work investigated the threshold beyond which characters are perceived as real, by morphing between photographs and puppet faces^16^ or photographs and virtual faces^17^. Despite its intuitive appeal, the uncanny valley concept still lacks empirical support^18^ and no final agreement exists about the measurement scales^19^ or potentially underlying perceptual mechanisms. However, facial features such as skin impurities^20^ or strong deviations of facial proportions like too big eyes^21^ have been identified as reliable characteristics of unappealing characters.

Event-related potentials (ERPs) are an excellent way to analyse face perception, as the measured brain responses can distinguish between highly automatic and more controlled stages of processing and do not require overt responses that may be distorted by experimental demand characteristics. Neuroscientific research has shown that face processing strongly activates dedicated areas in the visual cortex, in particular the occipital face area^22^,^23^ and the fusiform face area^24^. The fusiform face area is also involved in generating the face-sensitive N170 component in ERP studies^25^. Previous work showed that the N170 component peaks selectively for faces^26^. Real faces elicit a stronger N170 compared to abstract sketches of faces, but the difference is not statistically significant compared to schematic faces^27^, suggesting a gradual increase. Larger N170 amplitudes are found for human faces compared to those of other species^28^. Attractiveness also seems to induce small modulations in the N170 for characters of the same stylization level^29^,^30^. Further, it has been found that baby faces cause a higher N170 amplitude than adult faces, most likely due to neotenic features, like proportionally larger eyes^31^. Finally, a recent meta-analysis indicated that larger N170 amplitudes are observed for angry, fearful, and happy than for neutral facial expressions^32^. Moreover, emotion effects at the N170 can be found for faces of medium stylization^33^ and even for robots with rather schematic faces^34^.

Enhanced processing of emotional faces is also reflected in an enhanced Early Posterior Negativity (EPN), as well a larger Late Positive Potential (LPP)^35^,^36^. The EPN indicates early attention mechanisms, whereas LPP is viewed as reflecting higher-order evaluation and episodic memory encoding (for an overview see ref. 37). Both EPN and LPP amplitudes are sensitive to voluntary attention deployment to a stimulus and can be increased by explicit attention instructions^38^,^39^,^40^. Both ERP components are also sensitive to emotional content of various stimulus types, including faces^41^,^42^,^43^,^44^,^45^. This emotion sensitivity is thought to reflect intrinsically motivated attention to emotional stimuli and often co-varies with stimulus intensity or arousal^35^,^46^,^47^,^48^. As, in the absence of relevant social context, participants typically report less subjective arousal for picture of emotional faces than for pictures of emotional scenes, differences in arousal-level might account for generally smaller ERP emotion effects in response to faces compared to scenes^49^.

Apart from emotional expressions, larger EPN^50^ and LPP amplitudes^30^,^50^ are reported for attractive faces compared to unattractive faces, also applying to moderate stylizations^29^. Interestingly, in a recent study contrasting real neutral faces with neutral faces of puppets, no differences at the N170 level were observed, while from 400 ms onwards a larger LPP was found for real faces^51^. This was attributed to the salience and unique identity of a real face and to mentalizing about the depicted individual^51^, as generally computer generated faces are harder to remember^52^,^53^. In this vein, manipulating perceived uniqueness or distinctiveness via shape or reflectance manipulations of initially non-distinctive real faces has been found to result in a larger late positivity as well as a better memory performance^54^,^55^. Further, biographical information facilitates the processing of real faces at late ERP components^56^,^57^, which has recently also been shown for realistic computer generated faces^58^. The combined evidence suggests that a unique identity, either pre-existing or ascribed, enhances processing particularly at late stages.

Against the above background, the present research systematically addresses how brain responses known to reflect distinct stages of face processing vary across different levels of character stylization and for different emotional expressions. While previous work, in general, compared a real photograph of a person to a single stylization level, no previous study systematically manipulated face stylization of the same identity and studied how this influences brain responses towards emotional and neutral expressions. Our stimulus set exhibits six levels of stylization for two identities, depicting happy, neutral and angry expressions^20^. These cover a wide range of stylization, were professionally created, and match quality and style of current movies. All stimuli are rendered under the same conditions, thus reducing inconsistencies to a minimum. We expected to find strongest responses for real faces at the N170, EPN and LPP^26^. For very abstract faces the proportionally larger eyes and uniformly coloured textures might also cause a strong N170^31^. For angry and happy faces, we expected to find larger N170, EPN and LPP amplitudes. Interactions between level of stylization and emotional expression were explored on all components. Moreover, source analyses were employed to uncover the cortical generators of stylization effects, addressing the possibility of differential recruitment of visual areas such as fusiform or occipital face areas.

## Results

### ERP results

#### N170

**Figure 1:**
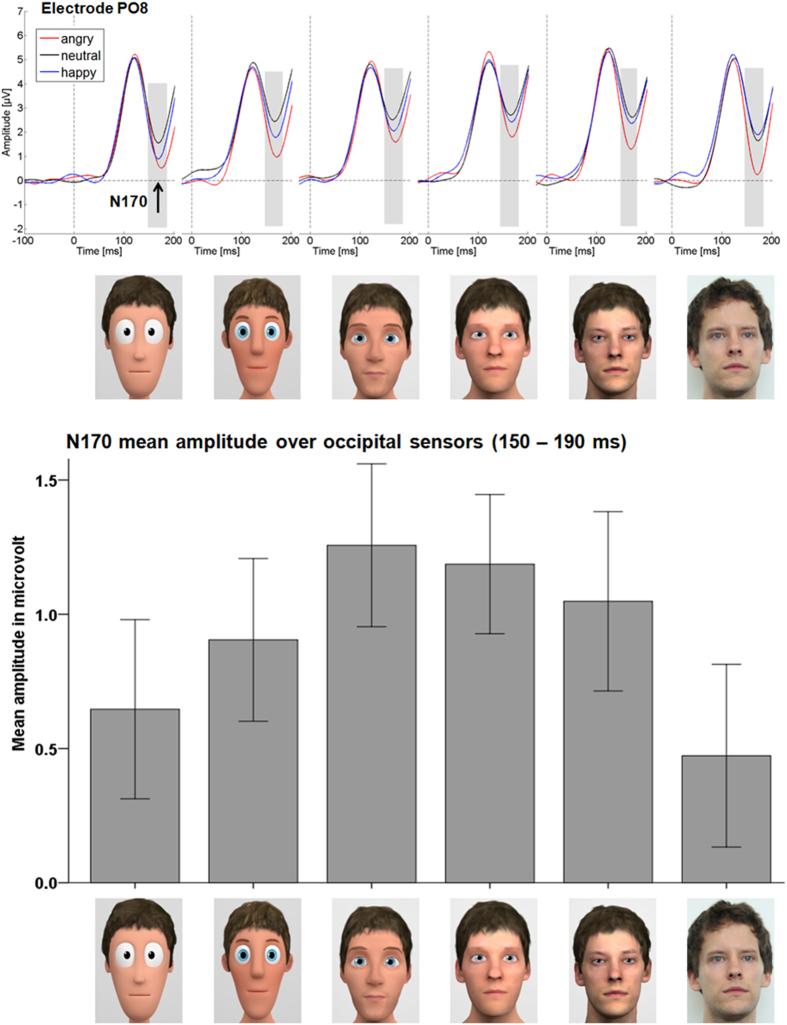
N170 ERP results. The upper panel displays the N170 at electrode PO8. From this panel, the interaction between emotion and realism can be observed. The lower panel shows the mean N170 over the occipital sensor cluster. Error bars are +/− one standard error of the mean. Note that, while negative-going, the N170 peak is still in the positive range (see top panel). Therefore, smaller bars represent higher N170 amplitudes.

**Figure 2:**
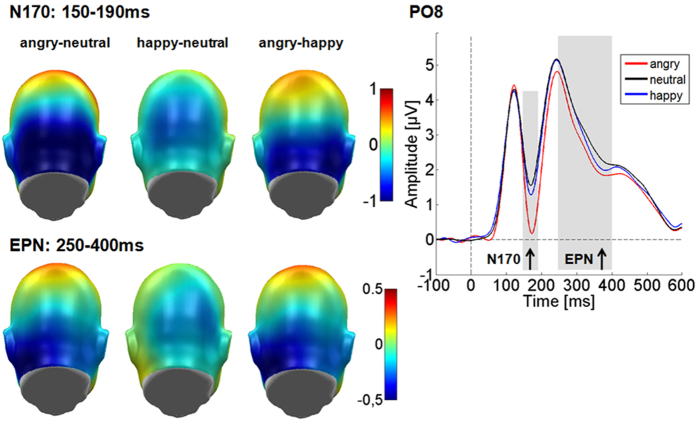
Emotion main effects for the N170 and the EPN. Left: Displayed are the difference topographies for the main effects of emotion across all stylization levels. Blue colours indicate a relatively larger negativity and red colours a larger positivity. Right: Displayed is the time course at electrode PO8.

For the N170 component significant modulations of stylization (*F*_(5,160)_ = 15.93, *p* < 0.001, partial η_P_^2^ = 0.332; see Fig. 1), emotion (*F*_(2,64)_ = 50.33, *p* < 0.001, partial η_P_^2^ = 0.611; see Fig. 2), as well as an interaction of stylization with emotion (*F*_(10,320)_ = 2.44, *p =* 0.008, partial η_P_^2^ = 0.071) were found over the two large symmetrical occipital sensor groups (see Fig. 1). For stylization levels, we tested a linear (*F*_(1,32)_ = 0.09, *p =* 0.765, partial η_P_^2^ = 0.003), compared to a u-shaped, quadratic, contrast (*F*_(1,32)_ = 68.50, *p* < 0.001, partial η_P_^2^ = 0.682), and found a u-shaped form for the face-specific N170 component. Although these u-shaped forms are visible and highly significant for all emotions, we identified with increased realism less intense reactions towards happy expressions and more intense reactions towards angry faces (see Fig. 1, upper panel). Moreover, emotional modulations resulted in a stronger quadratic (*F*_(1,32)_ = 59.81, *p* < 0.001, partial η_P_^2^ = 0.651), compared to a linear contrast (*F*_(1,32)_ = 41.64, *p* < 0.001, partial η_P_^2^ = 0.565), showing the most pronounced N170 for angry faces, smallest for neutral faces and in between happy expressions (all *ps* < 0.001).

Additionally, there was a trend for a main effect of channel group (*F*_(1,32)_ = 3.37, *p =* 0.075, partial η_P_^2^ = 0.095) and an interaction of stylization with channel group (*F*_(5,160)_ = 3.30, *p =* 0.025, partial η_P_^2^ = 0.087), showing in tendency a u-shaped distribution with two maxima over the left and a u-shaped distribution with one maximum over the right sensor cluster. The interaction of emotion with channel group (*F*_(2,64)_ = 0.58, *p =* 0.532, partial η_P_^2^ = 0.018) and triple interaction were both insignificant (*F*_(10,320)_ = 1.03, *p =* 0.471, partial η_P_^2^ = 0.031).

#### EPN

In the EPN time range, over the same cluster, main effects of emotional content (*F*_(2,64)_ = 19.89, *p* < 0.001, partial η_P_^2^ = 0.383) and laterality were observed (*F*_(1,32)_ = 33.83, *p* < 0.001, partial η_P_^2^ = 0.514; see Fig. 2). The EPN effect was somewhat better explained by a u-shaped form (*F*_(1,32)_ = 22.73, *p* < 0.001, partial η_P_^2^ = 0.415), compared to a linear contrast (*F*_(1,32)_ = 17.59, *p* < 0.001, partial η_P_^2^ = 0.355), where the strongest responses were found for angry faces, least responses to neutral faces and happy faces in between. The effect of laterality showed a larger EPN on the right compared to the left sensor group.

There was no effect of stylization (*F*_(5,160)_ = 1.56, *p =* 0.215, partial η_P_^2^ = 0.046), and no interaction of stylization with emotion (*F*_(10,320)_ = 1.04, *p =* 0.412, partial η_P_^2^ = 0.031), or channel group (*F*_(5,160)_ = 0.80, *p =* 0.555, partial η_P_^2^ = 0.024). Further, there was no interaction of emotion with channel group (*F*_(2,64)_ = 0.76, *p =* 0.474, partial η_P_^2^ = 0.023) and no triple interaction (*F*_(10,320)_ = 1.59, *p =* 0.108, partial η_P_^2^ = 0.047).

#### LPP

**Figure 3:**
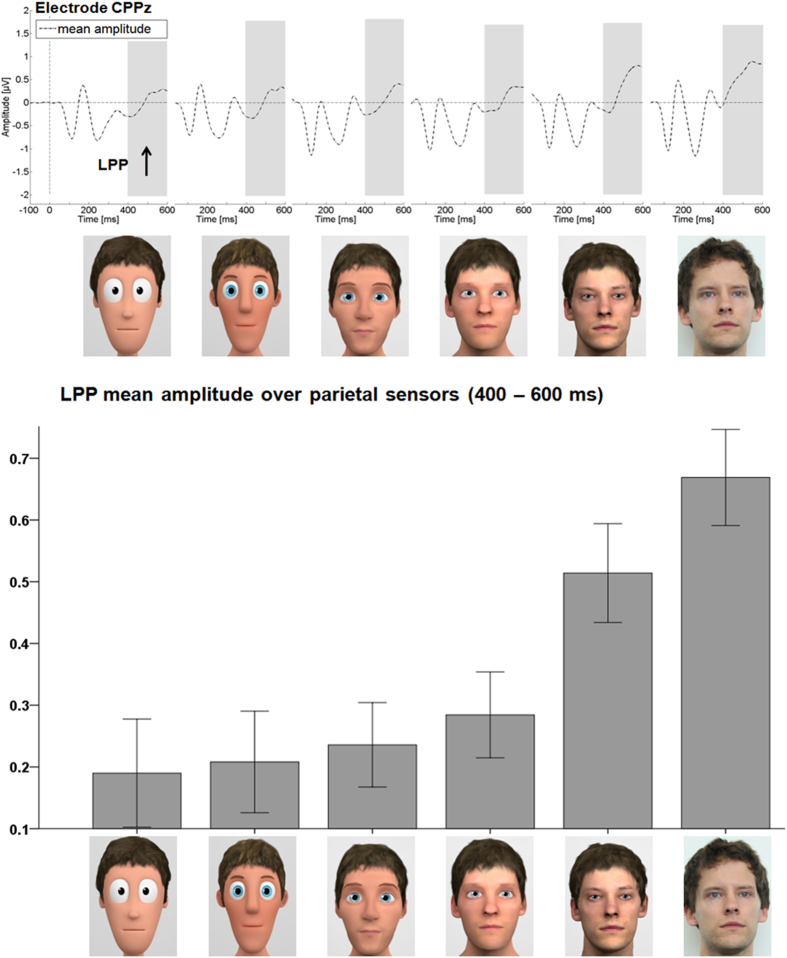
LPP ERP results. The upper panel shows the time course for electrode CPPz. Here an increase of LPP amplitudes can be seen for increasing realism. The lower panel displays the mean LPP over the parietal sensor cluster. Error bars are +/− one standard error of the mean.

In the LPP time window, over a large parietal sensor group, we found only a main effect of stylization (*F*_(5,160)_ = 12.62, *p* < 0.001, partial η_P_^2^ = 0.283). Here, a linear increase in the LPP amplitude was observed (see Fig. 3). The linear contrast (*F*_(1,32)_ = 31.28, *p* < 0.001, partial η_P_^2^ = 0.494) accounted for substantially more variance explained than did a u-shaped contrast (*F*_(1,32)_ = 11.39, *p =* 0.002, partial η_P_^2^ = 0.262).

There was no effect of emotion (*F*_(2,64)_ = 0.04, *p =* 0.965, partial η_P_^2^ = 0.001), and no interaction of stylization with emotion (*F*_(10,320)_ = 1.00, *p =* 0.444, partial η_P_^2^ = 0.030).

### Source reconstruction

**Figure 4:**
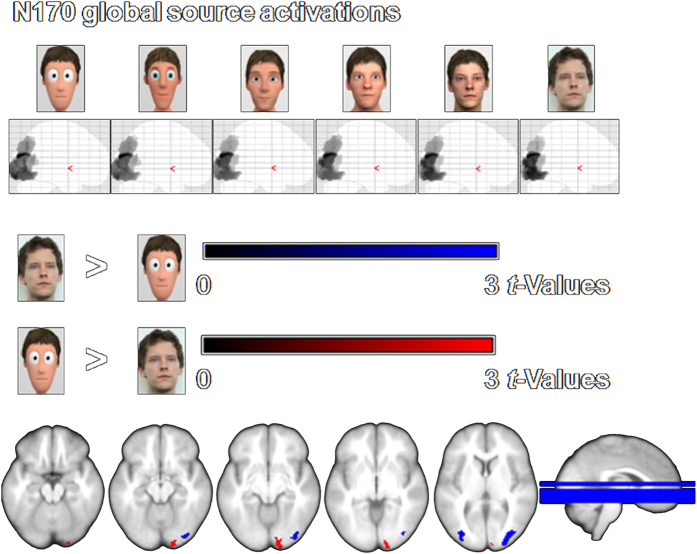
Source estimations for main effects of stylization level for the N170. Upper panel: Displayed are the family-wise error corrected (*p* < 0.05) changes in source activity for each stylization level. For all faces, significant generators can be observed for the N170 in bilateral superior, middle and inferior occipital gyri, as well as in the bilateral fusiform gyri. Lower panel: Displayed are the differences in source activity between stylization levels (post-hoc contrasts, uncorrected *p* < 0.005). In the N170 the real faces lead to larger middle and inferior occipital activations, while the most stylized faces are processed more intensely in the right inferior occipital gyrus/cuneus/lingual gyrus.

**Figure 5:**
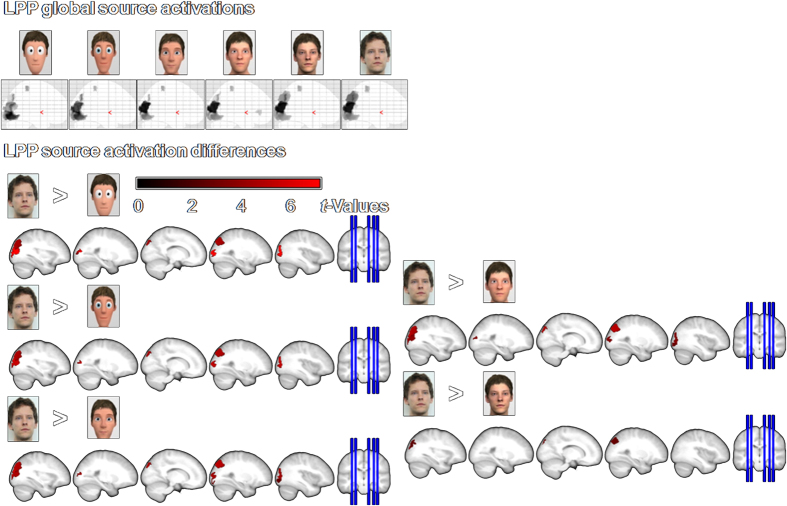
Source estimations for main effects of stylization for the LPP time window. Upper panel: Displayed are the family-wise error corrected (*p* < 0.05) changes in source activity for each realism level. For all faces, significant generators can be observed for the LPP in bilateral superior, middle and inferior occipital gyri, as well as in the bilateral fusiform gyri and bilateral superior parietal areas. Lower panel: Displayed are the differences between realism levels (post-hoc contrasts, uncorrected *p* < 0.005). In the LPP, real faces are processed more intensely in bilateral middle and superior occipital and superior parietal areas. However, with increased realism, these differences become smaller and finally disappear in middle occipital regions.

Source reconstructions were calculated for significant effects of stylization level. All presented faces elicited strong visual responses (see Figs 4 and 5 upper panel). However, the extreme poles (photographs compared to cartoon faces) showed a distinct processing already in the N170: While real faces led to larger inferior and middle occipital activations, highly stylized faces caused stronger responses in the right cuneus/lingual gyrus (see Fig. 4 and Table 1). Thus, despite similar N170 peaks for the extreme poles, the cortical generators differ. For neither of the extreme poles did we find statistical differences in source localization compared to moderately stylized characters.

Later, in the LPP stronger superior occipital and superior parietal activations are observed for real compared to cartoon faces (see Fig. 5 and Table 2). These source estimations mirror the linearly increasing LPP for higher realism. With increasing realism of the faces, the differences to real faces become smaller and finally disappear in middle occipital areas. For the reverse contrasts, no differences were found.

## Discussion

This study investigated the cerebral processing of stylized faces across six levels of realism and three levels of emotional expression (happy, angry, and neutral), as reflected by the N170, EPN and LPP components. The results demonstrate that character stylization affects both the N170 and the LPP amplitudes, albeit in a qualitatively different manner. For the N170, a u-shaped modulation was observed, while continuous amplitude increases with increasing realism occurred for the LPP. For the N170 both highly stylized faces and real faces elicited strongest responses. Further, at the N170 level, a differential effect of face stylization on emotional expression was found: For cartoon characters, happy expressions caused similar N170 amplitudes as did angry faces, while for realistic faces (levels 5 and 6) only angry expressions were selectively processed. The EPN component was modulated solely by emotional expression, angry faces eliciting largest amplitudes.

**Figure 6:**
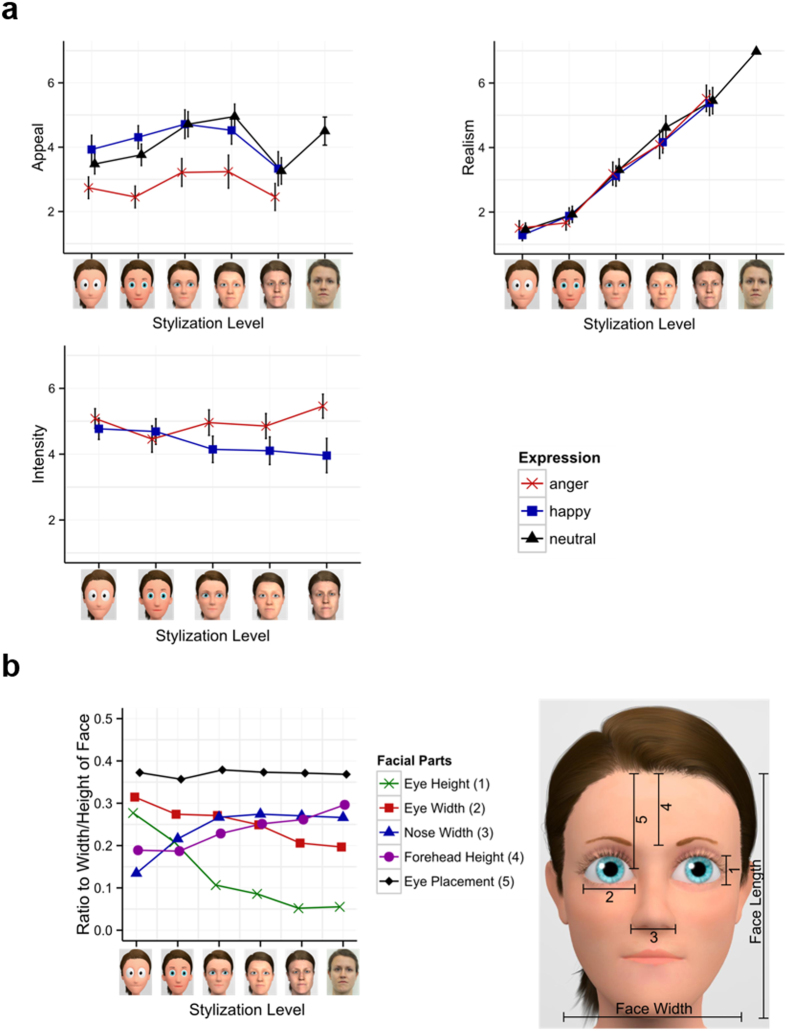
(**a**) Realism, appeal and intensity ratings from Zell *et al*.^20^, which tested the same characters but from slightly different point of view. Icons along the x-axis represent the stylization level. (**b**) Length and width of different facial parts, proportionally to the length and width of the face–averaged across both characters. For more stylized characters, the bigger eyes and smaller nose follow neotenic characteristics. However, this is not the case for vertical eye placement or forehead height. Error Bars denote 95% confidence levels.

The results are striking in that they reveal a dissociation of stylization effects on the N170 and LPP: Taking into account that (i) the N170 amplitude is larger for faces than for objects^24^,^25^,^31^, (ii) larger for real compared to schematic faces^27^ and (iii) cuteness and baby-like features have been associated with a larger N170^31^, we suggest neoteny and perceived face realism to drive the u-shaped N170 modulation. Analysis of stimulus properties (see Fig. 6) indicates that neotenic features, such as eye size, decrease non-linearly and very quickly for stylization levels 1 to 3. On the other hand, perceived realism increases linearly for the tested characters.

N170 generators were found to differ between very abstract and realistic faces: Although all faces activated extended visual regions, including the right fusiform gyrus, cartoon faces elicited stronger early visual cortex activations (cuneus, lingual gyrus, inferior occipital gyrus), while for real faces, stronger activations were found in middle occipital regions. Results suggest that processing of highly stylized faces relies more on structural analysis, associated with the so-called occipital face area, whereas realistic faces activate to a greater extent holistic processing, associated with the fusiform face area^6^. Fusiform responses have been found for a variety of face stimuli^59^. However, within computer generated characters, stronger fusiform responses were found when these looked and acted naturally and meaningfully^60^. The present U-shaped modulation could result from an interaction of perceived realism activating fusiform-dependent holistic processing and neoteny features activating feature-based processing in more occipital face areas.

Emotion effects on the N170 and EPN are in line with previous work^32^,^33^,^35^. They indicate that emotional expressions modulate the N170 and EPN responses across stylization levels, while, also in line with the literature, in real faces, angry expressions had the largest impact on ERPs^32^.

**Figure 7:**
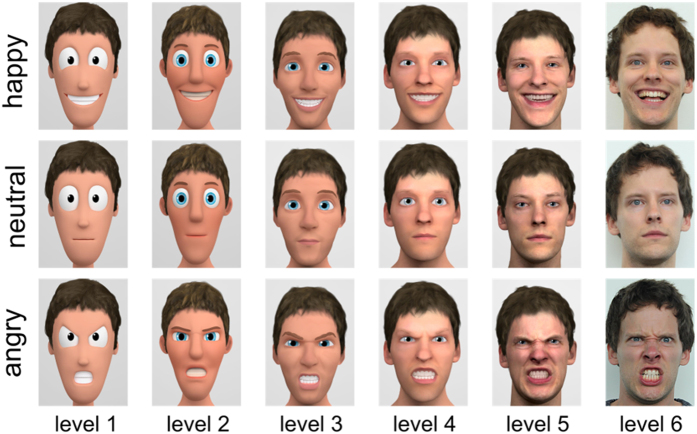
Example for emotional and neutral expressions for all six realism levels. The stimulus set consists of a male and female character. Here, the male character is shown for illustration purpose. Please refer to Fig. 6 for illustration of the female character.

It is remarkable that stylization and emotional expressions interact on the N170, indicating an early interplay of structural analysis and emotional classification, rather than dual processing routes for identity and expression. The present data suggest that with increasing realism more resources are captured by cues signalling threat^61^. Accordingly, more realistic angry faces were rated more intense compared to happy faces see Figs 6 and 7 and^20^. On the other hand, for very stylized faces, a relatively stronger processing of happy expressions was observed. Neotenic features may selectively enhance the processing of happy expressions. With the exception of the forehead, all neotenic features decrease or remain nearly unchanged with increasing realism (Fig. 6b). As big eyes and a small nose contribute highly to a cute perception of the character, possibly amplifying processing of positive expressions.

Unlike the N170 amplitude, LPP amplitude increase parallels perceived face realism across the tested stylization levels. This might be due to the uniqueness of a real face, prompting a multitude of ad hoc social inferences^62^. Bruce and Young’s influential model suggests that after initial perceptual and structural analyses, the observed stimuli are compared with face representations stored in memory and if there is a match, person-specific knowledge is retrieved^63^. Indeed, person-related semantic information enhances LPP amplitudes^56^. Recently, it has been further shown that biographical information can increase the LPP to computer-generated faces^58^. Although we did not provide participants with explicit biographical information, as a result of social inferences, the more realistic faces might be perceived as having a unique biographical identity. Behavioural evidence showed that computer generated faces are harder to remember, possibly because they are not encoded as a unique person^52^,^53^. The noticeable discontinuity between levels 1–4 and 5–6 could also imply a categorical change between realistic and non-realistic characters as shown by classification tasks at a similar stylization level^16^,^17^. Typically, distinctiveness is achieved by exaggerating certain spatial differences between an individual and an average face^64^. Distinctiveness by shape or reflectance manipulations has been found to result in a larger EPN and LPP as well as a steeper learning curve and better memory trace for initially non-distinctive faces^54^,^55^,^65^. However, naturally distinctive faces lead to the largest LPP and are remembered even better^55^. Regarding the creation of the currently used face stimuli, spatial differences were not overexaggerated in comparison to an average face. However, rated face-realism and distinctiveness might not be uncorrelated, as more realistic faces for example exhibit a more detailed texture. Other studies have related the enhanced LPP for real compared to doll faces to the unique identity of the real face, generating an impression of personal social presence^51^. Future studies should aim to disentangle effects of face-distinctiveness from face-realism.

Other factors can also modulate the LPP, but are unlikely to play a role in the current experiment: For instance, LPP responses increase with higher perceived familiarity^66^. However, Zell *et al*.^20^ report equal familiarity across the present stylization levels. Similarly, facial attractiveness enhances the LPP^30^,^50^. For our stimuli, appeal, which is conceptually similar to attractiveness, was rated highest for medium-stylized faces (see Fig. 6 and Zell *et al*.^20^). Therefore, it is unlikely that attractiveness is responsible for LPP modulations in our experiment. In source space, the linear modulation of the LPP was reflected in larger and broader activations in occipito-parietal areas. The localization of this increase is in line with both enhanced perceptual processing of more realistic faces and, in particular, also memory-related processes.

Although higher LPP amplitudes have been reported for emotional than for neutral stimuli e.g. refs 36, 41 and 44, we found no differences between emotional and neutral expressions on this component. In general, during passive viewing, emotion effects are smaller for faces compared to complex scenes and participants typically report less subjective arousal for faces^49^. Nevertheless, large emotion effects were present for the N170 and EPN time window. Similarly, Thom and colleagues^49^ found emotion effects for the N170, while for the EPN emotion effects were only descriptively visible and no differences were found for the LPP. This suggests that without an explicit task a highly automatic response towards emotional facial expressions modulates early components without affecting late stages of processing. In this vein, it has been shown that at late stages emotion effects benefit more from attention to the emotional category than do early responses^39^,^40^.

Overall, we demonstrated that stylized characters elicit neural effects that are different from the ones elicited by real faces. Importantly, the pattern changes qualitatively across different processing stages, although the measured facial features changed continuously across similar stylization levels. For face perception experiments, which use computer generated stimuli, this means that, unless a high level of realism is achieved, results cannot be transferred directly to real humans. Thus, computer generated stimuli may be suitable to test initial hypotheses, but require final validation with real photographs.

So far, it is unclear why or when exactly realism is beneficial in practical applications like games or perceptual studies, but the present study, in demonstrating that realism affects different processing stages in a distinct manner, may offer some clues: Both highly stylized faces, with their neotenic features and very realistic faces influence early stage processing and are equally efficient in transient attention capture. On the other hand, only more realistic faces induce the kind of post-processing necessary to build an individual identity representation and likely facilitating identification with the character. Beyond gaming, these findings have implications for the design of virtual reality therapy settings, for instance of social phobia. They underscore that depending on the overall goal optimal character design will differ. If so, the uncanny valley phenomenon may also arise from a perceived mismatch between situational expectations resulting from a given virtual scenario and character appearance.

In character design, the main problem for artists is that, for adult characters, neotenic and realistic features often exclude each other. For instance, skin smoothness is a neotenic feature, but detailed pores and skin-impurities are required to achieve full realism for a virtual character. Similarly, big eyes are considered as cute, but realistic characters that have unnaturally big eyes are perceived as creepy^21^, inverting the intended effect. This dichotomy between realistic characters on one side and rather cute characters on the other, could also explain the plausibility of the “uncanny valley” concept and the present data indeed reveal a neural dissociation that might support it. In practice, considering two independent scales–one for realism and one for neotenic features–seems to be a promising future direction to predict whether a stylized character will be perceived positively or negatively when used in game or as an interactive agent. Moreover, these parameters can be controlled more easily than appeal or attractiveness, which depend on many aspects and are more subjective.

As a limitation of the present work, it has to be noted that only two different identities were used. Therefore, our results might not generalize across all conceivable characters. In order to increase validity of the obtained results, our characters have been stylized based on popular 3D characters. Furthermore, the stimuli have been designed to match the quality of current animation movies as much as possible. To our best knowledge, our results are unique in that they provide many different stylization levels for the same characters.

## Conclusion

We measured EEG responses elicited by carefully manipulated faces with six different degrees of stylization and three emotional expressions. We tested a stimulus set for which a linear modulation of realism was achieved for the same identity. Our results indicate that face realism has a strong, but qualitatively different, influence on the N170 as well as the LPP component. While perceived realism influenced the N170 component in a u-shaped manner and interacted with emotional expression, the LPP component was only influenced by perceived realism, increasing continuously with face realism. For the N170, main generators differed between highly stylized and very realistic faces, suggesting to distinct contributing processes. The increased LPP was based on enhanced activity in broad occipito-parietal areas, in line with enhanced perceptual processing and memory encoding of more realistic faces.

## Methods

### Participants

Thirty-three participants were recruited at Bielefeld University. They gave written informed consent and received 11 Euros or course credit for participation. The study was approved by the Bielefeld University ethics committee (EUB number 2016-112). All methods were performed in accordance with the guidelines and regulation at Bielefeld University. The participants (22 females) were 23.30 years on average (*SD* = 3.68), all of them right-handed and had normal or corrected-to normal vision. Upon structured interview, no participant reported a current neurological or psychiatric disorder or relevant medication intake.

### Stimuli

Two face characters (one male, one female) varying across six stylization levels, with three emotional expressions (happy, angry, neutral) per stylization level, were used as stimuli. Creating continuous stylization of the same character is still an unsolved problem. While certain stylization effects can be achieved by image editing, using non-photorealistic rendering algorithms or character generators, all these approaches either do not modify the shape at all or address only a single stylization level. So far no automatic approach exists that accomplishes character stylization comparable to trained artists. Professional stylization is in general very time consuming and expensive. To circumvent this problem, most previous work used unrelated characters at different stylization levels. In contrast, Zell *et al*.^20^ published a set of stimuli that contains six stylization levels of the same person matching the state of the art in computer graphics. For completeness, we shortly report the stimuli creation process. Level 6 are real photographs. Models for level 5 have been created from high resolution 3D scans of the photographed people. These 3D scans have been post-processed to remove visible artefacts or to add hair. The remaining stylization levels have been created by professional artists targeting popular looks of animation movies. All emotions have been created so that specific features remain consistent (e.g., teeth shown consistently for happy and angry expressions). Levels 2, 3 and 5 were created first and evaluated in a study according to perceived realism and familiarity. All characters had very similar familiarity ratings as none of the characters is known to the public. In addition, for the initial characters, discontinuities were detected for rated face-realism. In order to achieve a stepwise increase in rated realism and sample the stylization scale more uniformly, level 1 and 4 have been created and added afterwards. The whole stimulus set was finally rated according perceived realism, appeal and expression intensity (Fig. 6). For the real photographs, only the neutral expression was rated in perceived realism and appeal. In addition, facial neotenic cues have been computed by measuring the relative size of facial parts^67^. We refer the reader to Zell *et al*.^20^ for a full description of the stimuli creation process, including all technical details and initial evaluation. For the current study, camera view and aspect ratio have been adjusted, such that all faces are nearly of the same size and the eyes are located at similar positions. Background planes with a 50% grey were inserted in the 3D scenes before rendering. For stimuli examples see the depicted faces in Figs 6 and 7.

### Procedure

The faces were randomly presented for 600 ms, followed by a fixation cross with variable latencies (randomly between 400 and 500 ms). All faces were repeated fifteen times, for a total number of 540 presented faces, while it was prevented that the same stimulus was presented twice in a row. Faces were presented on a 15.4-inch screen (Dell Latitude D830) with a 1600 × 1200 pixel resolution (image width: 800; height: 1142). The background colour was 50% grey (RGB color values: 128; 128; 128). The stimulus presentation lasted for about 10 minutes, while the whole session took approximately 50 minutes. Participants had no task but were instructed to attend to the presented faces, while moving as little as possible. Participants were encouraged to reduce their eye-movements by focusing on the fixation cross.

### EEG recording and analyses

EEG was recorded from 128 BioSemi active electrodes (www.biosemi.com). Recorded sampling rate was 2048 Hz. During recording Cz was used as reference electrode. Biosemi uses two separate electrodes as ground electrodes: A Common Mode Sense active electrode (CMS) and a Driven Right Leg passive electrode (DLR). The two electrodes form a feedback-loop which enables to measure the average potential close to the reference in the AD-box (see http://www.biosemi.com/faq/cms&drl.htm, where also information about extra functions of the CMS/DRL loop can be retrieved). Four additional electrodes (EOG) measured horizontal and vertical eye-movement. These were placed at the outer canthi of the eyes and below the eyes.

Pre-processing and statistical analyses were done using BESA^®^ (www.besa.de), EMEGS^68^ and SPM8 for EEG data (http://www.fil.ion.ucl.ac.uk/spm/). Offline, data were re-referenced to the average reference and then filtered with a forward 0.16 Hz high-pass and a zero-phase 30 Hz low-pass filter. Filtered data were segmented from 100 ms before stimulus onset until 600 ms after stimulus presentation. The 100 ms before stimulus onset were used for baseline correction. Eye-movements were corrected using the automatic eye-artefact correction method implemented in BESA^69^. Additionally, trials exceeding a threshold of 120 μV were rejected. Overall, 4.04 percent of all electrode measurements were interpolated. Per participant, on average 5.41 percent of all trials were rejected, leaving 28.38 trials per cell, leading to 85 trials per realism condition and 170 trials per emotion condition.

Cortical source reconstructions of significant ERP differences were generated and statistically assessed with SPM8 for EEG^70^, following recommended procedures. First, a realistic boundary element head model (BEM) was derived from SPM’s template head model based on the Montreal Neurological Institute (MNI) brain. Electrode positions were then transformed to match the template head, which is thought to generate reasonable results even when individual subjects’ heads differ from the template^71^. Average electrode positions as provided by BioSemi were co-registered with the cortical mesh template for source reconstruction. This cortical mesh was used to calculate the forward solution. For the inverse solution, the group inversion algorithm was used^70^ and the solution was calculated from 100 ms pre-baseline to 600 ms after stimulus onset.

### Statistical analyses

EEG scalp-data were statistically analyzed with EMEGS. Six (stylization: level 1, level 2, level 3, level 4, level 5, level 6) by three (emotional display: angry, neutral, happy) repeated measure ANOVAs were set-up to investigate main effects of the communicative sender, emotion and their interaction in time windows and electrode clusters of interest. Partial eta-squared (η_P_^2^) was estimated to describe effect sizes, where η_P_^2^ = 0.02 describes a small, η_P_^2^ = 0.13 a medium and η_P_^2^ = 0.26 a large effect^72^. When Mauchly’s Test detected a violation of sphericity, degrees of freedom were corrected according to Greenhouse-Geisser. For readability, the original degrees of freedom but corrected p-values and effect sizes are reported. For significant main effects linear compared to U-shaped contrasts were calculated. Time windows were segmented from 150 to 190 ms to investigate the N170, from 250 to 400 ms to investigate the EPN, and from 400 to 600 ms to the LPP component, after collapsing all conditions and visual inspection of the ERP components. For the N170 and EPN time windows, two large symmetrical temporo-occipital clusters of thirteen electrodes each were examined (left: I1, OI1, O1, POO3, PO9, PO9h, PO7, PO7h, P9, P9h, P7, TP9h, TP7; right: I2, OI2, PO10, POO4, PO10, PO10h, PO8, PO8h, P10, P10h, P8, TP10h, TP8). For the LPP time windows a large parietal cluster was investigated (twenty-six electrodes: CCPz, CP5, CP5h, CP3, CP1, CPz, CP2, CP4, CP6, CPP5h, CPP3, CPPz, CPP4, P3, P1, Pz, P2, P4, PPO3, PPO1, PPOz, PPO2, PPO4, PO3, POz, PO4). Results did not change qualitatively, when selecting different literature-based electrode clusters for the N170, EPN or LPP^34^,^36^,^54^.

Source reconstructions were performed for the main effects of face-stylization. For each analyzed time window in scalp space, three-dimensional source reconstructions were generated as NIFTI images (voxel size = 2 mm · 2 mm · 2 mm). These images were smoothed with a Gaussian kernel using an 8 mm full-width half-maximum. The statistical comparisons used in source space were based on significant differences on the scalp. In line with previous studies^39^,^42^,^73^, we describe statistical differences in source activity of voxels differing at least at an uncorrected threshold of *p* < 0.005 and a minimum of twenty-five significant voxels per cluster. Additionally, in all tables results are shown applying a family-wise error corrected threshold of *p* < 0.05. The identification of activated brain regions was performed using the LONI atlas^74^.

## Additional Information

**How to cite this article:** Schindler, S. *et al*. Differential effects of face-realism and emotion on event-related brain potentials and their implications for the uncanny valley theory. *Sci. Rep.* **7**, 45003; doi: 10.1038/srep45003 (2017).

**Publisher's note:** Springer Nature remains neutral with regard to jurisdictional claims in published maps and institutional affiliations.

## Figures and Tables

**Table 1:** Source estimations for stylization level main effects for the N170 (150–190 ms).

Cluster-level	peak-level		MNI coordinates			LONI
Number of significant voxels	Peak *t* (1, 192)	Peak *p*-unc	x (mm)	y (mm)	z (mm)	area
*Level 6 > Level 1*						
408	3.35	<0.001	28	−90	2	Mid Occipital G R
86	2.70	<0.005	−34	−90	8	Mid Occipital G L
*Level 1 > Level 6*						
169	3.31	<0.001	18	−98	−14	Inf Occipital G R

Results show differences only between very stylized faces (Level 1) and real faces (Level 6). Real faces elicited more activity in middle occipital regions, while cartoon faces elicited more activity in the right inferior frontal gyrus/cuneus/lingual gyrus. Notes. No. of sig. voxel = the number of voxel which differ significantly between both conditions. Peak *p*-unc = uncorrected *p* value. For each significant peak, respective coordinates (x, y and z) are displayed in MNI space. If a cluster exhibited more than one peak, only the largest peak is reported. Area = peak-level brain region as identified by the LONI atlas. R/L = laterality right or left. G = Gyrus; Mid = middle.

**Table 2:** Source estimations for stylization level main effects for the LPP (400–600 ms).

Cluster-level	Peak-level		MNI coordinates			LONI
Number of significant voxels	Peak *t* (1, 192)	Peak *p*-unc	X (mm)	y (mm)	z (mm)	area
*Level 6 > Level 1*						
1331 (707^a^)	7.47	<0.001	−36	−86	18	Mid Occipital G L
700 (483^a^)	7.09	<0.001	28	−90	2	Mid Occipital G R
673 (209^a^)	4.74	<0.001	20	−82	30	Sup Occipital G R
*Level 6 > Level 2*						
1192 (639^a^)	6.51	<0.001	-38	−84	20	Mid Occipital G L
617 (356^a^)	5.51	<0.001	34	−88	16	Mid Occipital G R
685 (301^a^)	4.94	<0.001	20	−82	30	Sup Occipital G R
*Level 6 > Level 3*						
1215 (610^a^)	6.06	<0.001	−36	−86	20	Mid Occipital G L
684 (345^a^)	5.13	<0.001	20	−82	30	Sup Occipital G R
749 (53^a^)	4.98	<0.001	34	−92	2	Mid Occipital G R
*Level 6 > Level 4*						
1167 (348^a^)	5.63	<0.001	−34	−88	20	Mid Occipital G L
705 (369^a^)	5.24	<0.001	20	−82	30	Sup Occipital G R
565	4.10	<0.001	36	−86	0	Mid Occipital G R
*Level 6 > Level 5*						
243	3.18	<0.001	−38	−82	22	Mid Occipital G L
329	2.91	<0.005	20	−82	30	Sup Occipital G R

Results show enhanced activity for real faces compared to stylized faces. Real faces (Level 6) elicited more activity in middle and superior occipital regions. Differences become smaller with increasing realism of the stylized faces. Notes. ^a^Resulting cluster size with FWE-corrected threshold of *p* < 0.05 (≥25 significant voxels). No. of sig. voxel = the number of voxel which differ significantly between both conditions. Peak *p*-unc = uncorrected *p* Value. For each significant peak, respective coordinates (x, y and z) are displayed in MNI space. If a cluster exhibited more than one peak, only the largest peak is reported. Area = peak-level brain region as identified by the LONI atlas. R/L = laterality right or left. G = Gyrus; Inf = inferior, Mid = middle, Sup = superior.
